# 30 Hz Transcutaneous Auricular Vagus Nerve Stimulation Alleviates Abdominal Pain by Modulating EEG Activity in the α Frequency Band of the Brain

**DOI:** 10.1111/cns.70641

**Published:** 2025-10-30

**Authors:** Qinxian Huang, Qihan Li, Huan He, Qingchuan Zhao, Kai Yuan, Suping Cai

**Affiliations:** ^1^ Xi'an International Medical Center Hospital Xi'an Shaanxi People's Republic of China; ^2^ School of Life Sciences and Technology Xidian University Xi'an Shaanxi People's Republic of China; ^3^ National Clinical Research Center for Digestive Diseases and Xijing Hospital of Digestive Diseases, Xijing Hospital Air Force Medical University Xi'an Shaanxi People's Republic of China

**Keywords:** dorsomedial prefrontal cortex, EEG, lateral prefrontal cortex, persistent abdominal pain, transcutaneous auricular vagus nerve stimulation, α frequency band

## Abstract

Persistent abdominal pain (PAP) is linked to reduced prefrontal alpha oscillations, correlating with pain severity. Our study found that 30 Hz transcutaneous auricular vagus nerve stimulation (taVNS) effectively enhanced these alpha rhythms in key prefrontal regions and was selected for intervention. A 20‐day 30 Hz taVNS treatment significantly alleviated pain and promoted the normalization of brain activity, demonstrating its potential as a non‐pharmacological therapy for PAP.

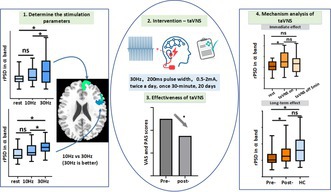

To the Editor,

Persistent abdominal pain (PAP), characterized by chronic discomfort localized between the thoracic and pelvic regions, is associated with significantly diminished quality of life, emotional distress, and heightened pain sensitivity, factors that may predispose individuals to developing comorbid chronic pain conditions [1]. Current clinical treatment of PAP faces challenges, as patients frequently experience suboptimal therapeutic outcomes or medication‐related adverse effects due to non‐specific pharmacological targeting [2]. Transcutaneous auricular vagus nerve stimulation (taVNS), a neuromodulatory approach, elicits analgesic effects through electrical activation of the auricular vagal nerve branch innervating the cymba conchae [3]. Notably, research investigating taVNS for refractory abdominal pain remains scarce, except for our recent study with a clinical trial demonstrating taVNS‐mediated amelioration of abdominal pain [4]. Given the extensive vagal innervation of gastrointestinal organs [5], taVNS emerges as a potential therapeutic intervention for PAP.

Twenty‐four PAP patients were recruited from Xi'an International Medical Center Hospital with abdominal pain persisting for over one year without an identifiable organic cause based on Rome IV and DSM 5. To address potential confounding effects from medication use for abdominal pain, the study implemented three measures (See Supporting Information). Twenty‐eight healthy controls (HCs) are from open recruitment. All participants were provided with information about the stimulation procedure, and wrote the informed consents. Firstly, the abnormal EEG characteristics of the PAP group compared with the normal group were detected. There is an effect of stimulation parameters of high frequency or low frequency on the intervention effect [6]. Thus, the present study was based on the changes in a specific frequency band of the EEG characteristics in normal subjects to assess the effect of two different stimulation parameters on this band in real‐time taVNS (taVNS on) (Figure 1C,E). A study has revealed an inverted‐U relationship between VNS stimulation frequency and induced cortical plasticity, demonstrating the highest cortical plasticity at a stimulation frequency of 30 Hz VNS [7]. Our verification experiments confirmed that 30 Hz is more effective for the abnormal areas identified in the PAPs (Figure 1D). Subsequently, a 30 Hz stimulation frequency with pulse widths of 200 μs was selected for taVNS (tVNS501, Ruishenan Medical Co. Ltd) clinical intervention in PAPs. The pulse amplitude intensity was adjusted based on the participants' tolerance (0.5–2 mA). Each patient was treated with taVNS twice a day (30 min each time, 12 h between the twice) at the cymba conchae of the left ear for 20 consecutive days (Figure 1F). The visual analog scale (VAS) and pain anxiety score (PAS) were scored and brain EEG was recorded before and after taVNS intervention. Detailed EEG processing information was given in Supporting Information (See Supporting Information).

**FIGURE 1:**
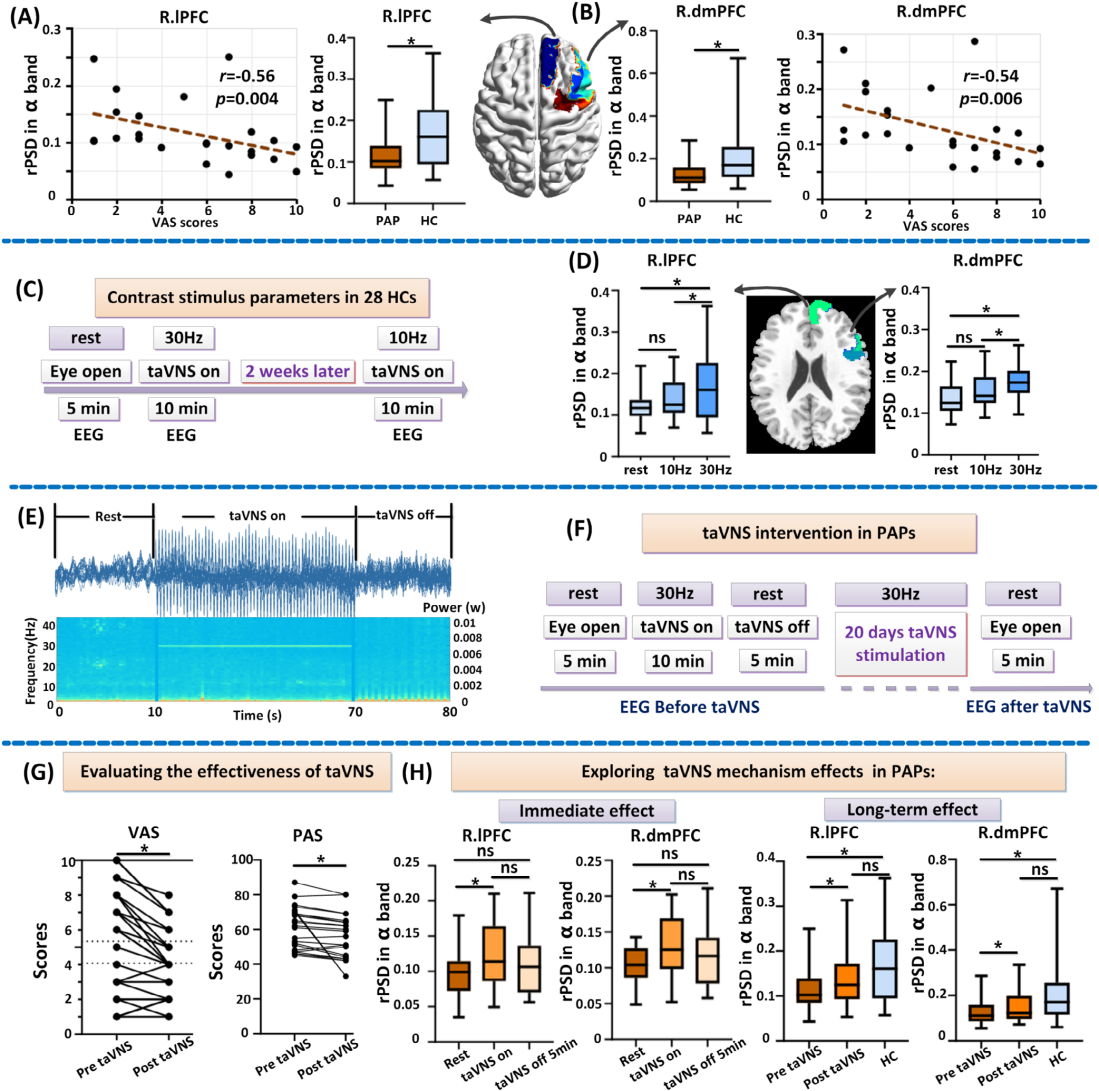
Study protocol and main results. (A, B) Detecting the abnormal EEG features in 24 PAPs: The rPSD in α band of R.lPFC and R.dmPFC was significantly reduced in PAPs and was significantly negatively correlated with the VAS scores; (C) Determining taVNS stimulus parameters in 28 HCs to assess the effect of the 30 Hz and 10 Hz stimulation parameters in real‐time taVNS; (D) 30 Hz stimulation demonstrated superior modulation amplitude compared to 10 Hz stimulation on the abnormal areas (R.lPFC and R.dmPFC) which were detected in the PAPs; (E) Example image of power changes under different frequency in three states EEG; (F) 20 days taVNS intervention in 24 PAPs; (G) Evaluating the effectiveness of taVNS (30 Hz) in 24 PAPs; (H) Exploring taVNS mechanism effects in PAPs including immediate and long‐term effects combining 10 min real‐time taVNS (taVNS on). *represents *p* < 0.05 (FDR correction); ns: Not statistically significant. R.dmPFC, right dorsomedial prefrontal cortex; R.lPFC, right lateral prefrontal cortex.

Our analysis revealed significantly decreased α‐band (8–13 Hz) relative power spectral density (rPSD) in the right lateral prefrontal cortex (R.lPFC) and right dorsomedial prefrontal cortex (R.dmPFC) of PAP patients compared to HCs (Figure 1A,B). The rPSD of the R.lPFC and R.dmPFC was significantly negatively correlated with the VAS scores (Figure 1A,B). It suggested the clinical significance of these two regions. To optimize α‐frequency modulation in these two regions, we compared 30 Hz to 10 Hz stimulation efficacy in the HC cohort (Figure 1C,E). Notably, 30 Hz stimulation demonstrated superior modulation amplitude compared to 10 Hz stimulation (Figure 1D). Therefore, we adopted 30 Hz for taVNS intervention (Figure 1F). Following a 20‐day intervention protocol, PAP patients exhibited significant reductions in both VAS and PAS scores (Figure 1G), although there was no correlation between changes in VAS pain scores and changes in anxiety levels (Figure S1). Concurrently, rPSD values in the targeted prefrontal regions showed a marked elevation, approaching the normative levels observed in HCs. EEG was recorded at resting‐state, during taVNS‐on, and 5 min post‐stimulation (taVNS‐off). taVNS enhanced α‐rPSD, and this enhancement persisted post‐stimulation, showing no significant difference from either the taVNS‐on or resting‐state conditions (Figure 1H). This indicates that the immediate effects of taVNS are subtherapeutic and that cumulative stimulation is required for efficacy.

taVNS has gained recognition as a promising neuromodulatory intervention for conditions including irritable bowel syndrome (IBS), functional gastrointestinal disorders, and primary dysmenorrhea, with demonstrated efficacy in reducing pain intensity and mechanical pain sensitivity [8, 9]. Despite this progress, evidence for taVNS in treating PAPs remains limited. To our knowledge, this study represents the first therapeutic investigation of taVNS for PAP, specifically targeting vagus nerve modulation and evaluating real‐time EEG signals. Following a 20‐day taVNS stimulation, clinical pain metrics (VAS, PAS) of PAP patients have decreased to different degrees, highlighting the intervention's therapeutic potential. Beyond establishing the superior efficacy of 30 Hz stimulation over 10 Hz in pain alleviation, this work provides mechanistic insights through real‐time neurophysiological monitoring. Our findings suggest that taVNS‐mediated improvement in abdominal pain may involve normalization of α‐band oscillatory activity within the R.lPFC and R.dmPFC. Real‐time EEG analysis revealed progressive enhancement of α‐band power during active stimulation, a rhythm associated with relaxed yet attentive states conducive to pain modulation. Thus, taVNS might be considered an option for auxiliary treatment in relieving abdominal pain. Patients with PAP are often associated with psychological distress and anxiety emotions, which may in turn amplify and maintain the pain state [10]. Through clinically observed changes in pain scores and anxiety scores, we speculate that taVNS might alleviate the perception of abdominal pain by relieving pain‐related anxiety. Due to the lack of a placebo control, we cannot determine whether the observed effects are specific to taVNS, which should be verified by establishing a rigorously designed sham stimulation control group in future studies. The sample size is another limitation in the detection of more subtle effects or complex interactions. To fully optimize taVNS, future work must not only test different parameter combinations (frequency, pulse width, intensity) in larger populations but also demonstrate their long‐term efficacy and safety for home use through robust follow‐up studies.

In conclusion, our findings suggest that 30 Hz taVNS has the potential to provide a non‐pharmacologic treatment option for PAP. Moreover, it could provide support to develop a home‐based therapeutic device to address broader clinical needs, providing convenient auxiliary treatment for more patients with abdominal pain.

## Author Contributions

**Qinxian Huang:** methodology. **Qihan Li:** writing – original draft. **Huan He:** data curation. **Qingchuan Zhao:** investigation. **Kai Yuan:** writing – review and editing. **Suping Cai:** supervision. All authors have read and approved the final draft submitted.

## Ethics Statement

Ethical statement was approved by the Xi'an international medical center hospital ethics committee. The clinical registration was approved (ChiCTR2500103017).

## Conflicts of Interest

The authors declare no conflicts of interest.

## Supporting information


**Figure S1:** The correlation between changes in VAS pain scores and changes in anxiety levels.

## Data Availability

The data that support the findings of this study are available from the corresponding author upon reasonable request.
