# Supplementary material for: 30 Hz Transcutaneous Auricular Vagus Nerve Stimulation Alleviates Abdominal Pain by Modulating EEG Activity in the α Frequency Band of the Brain
Source: CNS Neurosci Ther. 2025 Oct 30;31(11):e70641. doi: 10.1111/cns.70641 (PMC12575443; doi:10.1111/cns.70641)
Supplement: Supplementary file 1 — Figure S1: The correlation between changes in VAS pain scores and changes in anxiety levels. [file CNS-31-e70641-s001.docx]

**30 Hz transcutaneous auricular vagus nerve stimulation alleviates abdominal pain by modulating EEG activity in the α frequency band of the brain**

Qinxian Huang^1^(MD), Qihan Li^2^ (MD), Huan He^1^ (MD), Qingchuan Zhao^3^ (Ph.D), Kai Yuan^2,*^ (Ph.D) and Suping Cai^2,*^(Ph.D)

*^1^*^.^ *Xi’an International Medical Center Hospital, Xi’an, Shaanxi 710100, PR China;*

*^2.^ School of Life Sciences and Technology, Xidian University, Xi’an, Shaanxi 710071, PR China;*

*^3.^ National Clinical Research Center for Digestive Diseases and Xijing Hospital of Digestive Diseases, Xijing Hospital, Air Force Medical University, Xi'an, Shaanxi 710000, PR China;*

Supplementary catalogue

Methods and materials 1

01.Participants 1

02. taVNS application and experimental protocol 2

03. EEG recording and preprocessing 2

04. rPSD analysis based on EEG preprocessed data 3

05. Statistical analyses 3

06. Discussion on practical challenges and safety of taVNS applications. 4

07. Limitations 4

08. References 4

## Methods and materials

### 01.Participants

In this study, twenty-four patients with persistent abdominal pain (PAP) (14 males and 10 females; age: 27.96 ± 3.27 years) were recruited from the outpatient clinic. These patients had experienced abdominal pain for more than one year without any identifiable organic etiology, as defined by the Rome IV criteria and DSM-5. The PAP patients were enrolled from Xi’an International Medical Center Hospital (Xi’an, Shaanxi 710000, PR China). Ethical statement was approved by the Xi'an international medical center hospital ethics committee. The clinical registration was approved (ChiCTR2500103017). To avoid potential confounding effects from medication use for abdominal pain, our study implemented the following measures: 1) All participants were thoroughly screened prior to enrollment. Those with a recent history of medication use for abdominal pain (e.g., analgesics, antispasmodics, or other symptom-altering drugs) were excluded unless they had undergone a sufficient washout period; 2) For any participants who had previously used such medications, a mandatory 7-day washout period was enforced prior to the initiation of the taVNS intervention. This ensured that any residual pharmacological effects were minimized before baseline assessments and stimulation; 3) Throughout the taVNS intervention period, participants were instructed to refrain from using any medications aimed at relieving abdominal pain. Additionally, 28 healthy controls (HCs) (18 males and 10 females; age: 22.15 ± 1.26 years) were recruited through open advertisement. None of the participants were undergoing any medical treatment at the time of the study. All participants were provided with comprehensive information regarding the experimental procedures and gave written informed consent prior to participation.

### 02. taVNS application and experimental protocol

Transcutaneous auricular vagal nerve stimulation (taVNS) was administered using a portable stimulator (tVNS501, Changzhou Rishena Medical Device Co.). Stimulation was applied exclusively to the left ear; electrodes were positioned within the left external acoustic meatus to target the inner surface of the cymba conchae [1]. The stimulation parameters were set as follows (The taVNS device allows for the configuration of repeated stimulation mode parameters): an on-time of 60 seconds, an off-time of 10 seconds, a pulse width of 200 μs, a pulse frequency of 30 Hz, and an amplitude adjustable between 0.5 mA and 2 mA, tailored to each participant’s maximum tolerable threshold. For the at-home treatment sessions (30 minutes twice a day), the exact same “60s on / 10s off” cycle was applied repeatedly for the full 30-minute duration. Apart from the sensation of a stinging pain in the ears, there were no other adverse reactions.

Participants were seated in a quiet, temperature-controlled room maintained at 26 °C and instructed to remain relaxed throughout the procedure. Baseline resting-state electroencephalography (EEG) data were recorded prior to taVNS stimulation, during which participants maintained both eyes-open (EO) and eyes-closed (EC) conditions for 5 minutes each. Subsequently, all participants underwent 10 minutes of taVNS stimulation in the EO state, after which post-stimulation EEG data were acquired during an additional 5-minute EO recording.

### 03. EEG recording and preprocessing

EEG data were acquired using a 64-channel cap (NeuSen W, Changzhou Neuracle Technology Co., Ltd.) at a sampling rate of 1000 Hz and with a pass-band of 1–300 Hz. Of the 64 electrodes, 59 Ag/AgCl electrodes were arranged according to the international 10–20 system, while five additional channels (ECG, HEOR, HEOL, VEOR, VEOL) were excluded from subsequent analyses. Electrode impedances were maintained below 20 kΩ. All EEG data analyzed in this study were acquired under eyes-open conditions. Throughout the entire recording session—including both baseline and transcutaneous auricular vagus nerve stimulation (taVNS) periods—participants were explicitly instructed to keep their eyes open (simulate a normal living environment). Preprocessing was conducted using EEGLAB [2] in MATLAB (R2021a; MathWorks, USA). For computational efficiency, the raw EEG data were down-sampled to 500 Hz. The EEG data were re-referenced offline to an average reference using custom scripts compatible with EEGLAB. Although the hardware reference during acquisition was placed according to the international 10–20 system, we applied average re-referencing during preprocessing to achieve a more spatially neutral baseline.

The detailed descriptions of EEG preprocessing steps are as follows: 1) Raw data were first band-pass filtered between (0.5 Hz) (high-pass) and 40 Hz (low-pass) using a zero-phase finite impulse response (FIR) filter to remove slow drifts and high-frequency noise. 2) Resting state ocular and muscle artifacts were identified and removed using independent component analysis (ICA). Components correlated with ocular (blinks, saccades) and muscular artifacts were identified and rejected based on their topography, time course, and correlation with EOG/EMG channels if available. These identified artifactual components were removed. The EEG data was then reconstructed without these artifactual components, effectively subtracting the artifact contribution from the signal. 3) The continuous data were segmented into 2-second epochs. 4) Each epoch was baseline-corrected using the entire epoch mean. 5) Bad channels were identified based on abnormal variance and interpolated using spherical splines with EEGLAB's pop_interp function.

The presence of stimulation-related artifacts during the taVNS-on condition is a well-known technical challenge in EEG research involving electrical stimulation. We employed a multi-step, rigorous approach to identify and mitigate these artifacts to ensure the integrity of the neural signals. Firstly, The continuous EEG data were first visually inspected to identify and mark segments with clear, overwhelming electrical artifacts that rendered the neural signals unrecoverable. These marked segments were excluded from subsequent analysis. Secondly, the data were band-pass filtered between 1 and 50 Hz. The EEG recordings were segmented into epochs of 2000 ms for each condition (during- and post-stimulation), with baseline correction applied based on the mean value of each epoch. Thirdly, we applied independent component analysis (ICA). This was our primary method for isolating and removing stimulation artifacts. ICA successfully separated neural activity from non-neural sources, including the periodic electrical artifacts from taVNS. Components clearly identified as noise based on their topography, frequency spectrum, and timing were manually rejected. Finally, the effectiveness of this cleaning procedure was validated by comparing the power spectral density and time-domain signals before and after artifact removal. This confirmed the significant reduction of noise while preserving the physiological features of the EEG. We acknowledge that while this approach significantly reduces stimulation artifacts, it may not eliminate all noise. We have therefore interpreted the results from the taVNS-on condition with appropriate caution.

Source localization was performed using BrainStorm [3], an open-source software platform for analyzing brain recordings. A common head model and source model were employed across all participants. Forward modeling was conducted using the OpenMEEG boundary element method (BEM) [4], and the linearly-constrained minimum variance (LCMV) beamformer algorithm [5] was used to estimate EEG sources based on the data covariance matrix. Finally, the cortical surface was parcellated into 100 regions of interest (ROIs) according to the Schaefer-100-7net atlas for subsequent analysis.

### 04. rPSD analysis based on EEG preprocessed data

Power spectral density (PSD) analysis was performed using a standard fast Fourier transform (FFT). The PSD for each ROI was estimated using Welch’s method with a frequency resolution of 0.5 Hz. For each brain region, the PSD was computed across all segments, and the power within specific frequency bands was extracted. Relative PSD (rPSD) for each frequency band (Delta: 1–4 Hz, Theta: 4–8 Hz, Alpha: 8–13 Hz, Beta: 14–30 Hz, Gamma: >30 Hz) was calculated as the ratio of the normalized PSD within the corresponding band to the total power across the frequency range of interest.

### 05. Statistical analyses

Statistical analyses were conducted on all quantified data. Pre- and post-intervention group comparisons utilized paired t-tests, while between two groups contrasts employed independent-sample t-tests or permutation test (5000 iterations) depending on whether the data follows a normal distribution. If more than two groups or conditions were compared, one-way analysis of variance (ANOVA) was applied. To control the proportion of false positives among the significant results, we applied the false discovery rate (FDR) correction using the Benjamini-Hochberg procedure at a level of q < 0.05 for multiple comparisons correction. The relationship between the brain measures and behavioral scores was assessed using Pearson's correlation coefficient (r). We also Calculated the Cohen's d which is a statistical metric used to measure effect size. It is most commonly used to compare the mean differences between two groups. We obtained Cohen's d = 0.9. All statistical analyses were performed using GraphPad Prism 10.1.2 using the age as a covariate.

We conducted the correlation analysis between changes in VAS pain scores and changes in anxiety levels. However, there was no correlation between them (Figure S1).

**
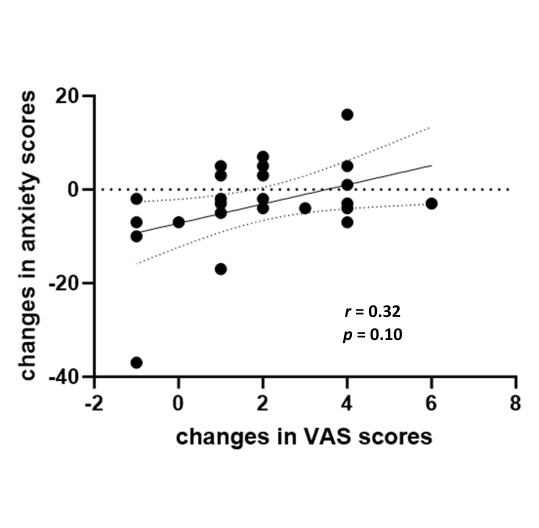
**Figure S1: The correlation between changes in VAS pain scores and changes in anxiety levels.

### 06. Discussion on practical challenges and safety of taVNS applications.

Possible challenges and safety regarding taVNS, we discussed the following aspects: 1) Individual variability differences in anatomy, such as the vagus nerve branching and ear morphology, and physiological baseline states may lead to variability in taVNS response, which presents a challenge for standardizing protocols. 2) While generally well-tolerated (e.g. apart from the sensation of a stinging pain in the ears, there were no other adverse reactions in our study), the sensation during stimulation may affect long-term adherence in some individuals, particularly in unsupervised, home-based settings. 3) The efficacy and safety of taVNS are likely dependent on specific stimulation parameters and device characteristics, highlighting the need for device standardization and certification for home use. 4) We have explicitly mentioned potential adverse effects such as skin irritation, discomfort, dizziness, or headache under the stimulation electrodes, based on previous literature and our own observations. We note that these effects were generally mild and transient in our study cohort. Or, for the majority of participants, they do not experience any discomfort, dizziness, or headache. 5) We conclude the paragraph by emphasizing the need for larger-scale, long-term safety studies, the development of user-friendly and safe home-based devices, and personalized strategies to optimize efficacy and maximize adherence.

### 07. Limitations

A limitation concerns the age difference between the PAP group and the HCs cohort. the healthy control (HC) group was significantly younger than the patient group. Although this cohort was instrumental in providing a normative benchmark for neuroplastic changes and we statistically adjusted for age by including it as a covariate in our analyses, this statistical control cannot fully compensate for the lack of an age-matched experimental design. The substantial mean age difference and the minimal overlap in age distributions mean that residual confounding is likely. Age is a proxy for broad neurophysiological changes that may influence EEG activity, and our model cannot account for all unmeasured, age-related factors. Therefore, while we observe promising trends of patient outcomes shifting toward the healthy profile post-treatment, these comparative results must be interpreted with caution. Future studies should prioritize recruiting an age-matched control group, potentially including both healthy individuals and a patient control group receiving sham stimulation, to conclusively isolate the treatment effects from demographic influences.

The discussion of clinical feasibility and safety considerations.1) Individual variability differences in anatomy, such as the vagus nerve branching and ear morphology, and physiological baseline states may lead to variability in taVNS response, which presents a challenge for standardizing protocols. 2) While generally well-tolerated (e.g. apart from the sensation of a stinging pain in the ears, there were no other adverse reactions in our study), the sensation during stimulation may affect long-term adherence in some individuals, particularly in unsupervised, home-based settings. 3) The efficacy and safety of taVNS are likely dependent on specific stimulation parameters and device characteristics, highlighting the need for device standardization and certification for home use. 4) We have explicitly mentioned potential adverse effects such as skin irritation, discomfort, dizziness, or headache under the stimulation electrodes, based on previous literature and our own observations. We note that these effects were generally mild and transient in our study cohort. Or, for the majority of participants, they do not experience any discomfort, dizziness, or headache.

### 08. References

[1] Shi X, Hu Y, Zhang B, Li W, Chen JD, Liu F. Ameliorating effects and mechanisms of transcutaneous auricular vagal nerve stimulation on abdominal pain and constipation. JCI Insight. 2021;6(14):e150052. Published 2021 Jul 22. doi:10.1172/jci.insight.150052

[2] Delorme A, Makeig S. EEGLAB: an open source toolbox for analysis of single-trial EEG dynamics including independent component analysis. J Neurosci Methods. 2004;134(1):9-21. doi:10.1016/j.jneumeth.2003.10.009

[3] Tadel F, Baillet S, Mosher JC, Pantazis D, Leahy RM. Brainstorm: a user-friendly application for MEG/EEG analysis. Comput Intell Neurosci. 2011;2011:879716.

[4] Gramfort A, Papadopoulo T, Olivi E, Clerc M. OpenMEEG: opensource software for quasistatic bioelectromagnetics. Biomed Eng Online. 2010;9:45. Published 2010 Sep 6.

[5]J aiswal A, Nenonen J, Stenroos M, et al. Comparison of beamformer implementations for MEG source localization. Neuroimage. 2020;216:116797.
